# A conceptual framework addressing the complex labour market dynamics of the work-to-retirement process

**DOI:** 10.1007/s10433-022-00704-3

**Published:** 2022-04-27

**Authors:** Taina Leinonen, Isabelle Boets, Elisabeta Pletea, Sofie Vandenbroeck, Ingrid Sivesind Mehlum, Hans Martin Hasselhorn, Astrid de Wind

**Affiliations:** 1 Finnish Institute of Occupational Health, Helsinki, Finland; 2 Environment and Health, Department of Public Health and Primary Care, KU Leuven, Leuven, Belgium; 3 Group IDEWE, Leuven, Belgium; 4 Multisector Occupational Health Service, Luxemburg, Luxemburg; 5 National Institute of Occupational Health (STAMI), Oslo, Norway; 6 Department of Occupational Health Science, University of Wuppertal, Wuppertal, Germany; 7 Amsterdam UMC Location University of Amsterdam, Public and Occupational Health, Amsterdam, The Netherlands; 8 Amsterdam Public Health Research Institute, Societal Participation and Health, Amsterdam, The Netherlands

**Keywords:** Theoretical model, Measurement, Employment, Labour force, Pension

## Abstract

The way in which retirement is conceptualized and measured is likely to influence the research findings. The previous literature has addressed a wide range of elements related to the complex work-to-retirement process, such as early, late and partial retirement, statutory retirement, work disability and unemployment paths to retirement, or different types of bridge employment. However, conceptual clarity in terms of connections between the different elements is called for. We introduce a conceptual framework of the work-to-retirement process to guide its future measurement. Together with information on the statutory retirement age, the main elements of the framework are based on employment and pension receipt, acknowledging that these may overlap. The framework is flexible to the user, providing the possibility to add various specifications—e.g. of types of employment, types of pension receipt, unemployment, and being outside the labour force—depending on the study context and aims. The framework highlights the complexity of the work-to-retirement process, bringing forth its multifaceted, multiphased and multidirectional features. Accounting for such complexity in later-life labour market dynamics helps to elaborate what is actually addressed when investigating “retirement”. Our conceptual framework can be utilized to enhance well-defined, precise and comparable measurement of the work-to-retirement process in studies.

## Introduction

Conceptualization and measurement of retirement is particularly relevant in ageing societies, where a growing number of studies have focused on the possibilities to extend working careers or increase the health and wellbeing of the large cohorts reaching retirement age. The concept of retirement generally relates to withdrawal from paid employment of workers who are relatively close to statutory retirement ages and typically entitled to pensions or other benefits. However, retirement is perceived as a highly complex phenomenon that may be multifaceted, multiphased, and even multidirectional. Therefore, retirement has often been conceptualized as a process instead of a single event (Beehr 1986; Denton and Spencer 2009; Wang and Shultz 2010; Beehr and Bennett 2015; Cahill et al. 2015; Hasselhorn and Apt 2015). Partly due to the complexity of the retirement concept, retirement is also measured in various ways (Hakola 2003; Denton and Spencer 2009). Ideally, prior conceptualization of retirement should guide the way in which it is measured. In practice, however, conceptualization and measurement of retirement may both be driven by the available data and may not always be clearly distinguished from each other in studies.

The ways in which retirement is conceptualized and measured, are likely to influence the research findings. In order to interpret and compare findings of retirement studies, the large variation in addressing retirement should be acknowledged and dealt with. Beehr (1986, p. 50) formulated the issue in the following way: “Retirement comes in several forms, and these forms may be preceded by different causes and may predict different outcomes. Exactly what the different studies are investigating will be made more evident by clearly defining retirement and its operationalizations.”

The primary aim of this article is to build on the previous literature to develop a conceptual framework of the work-to-retirement process that would guide its future measurement. Retirement can be conceptualized at institutional, organizational and individual levels (Szinovacz 2012). Our work adds to the individual-level conceptualization of retirement, which can further be considered in terms of *psychological* and/or *labour market* processes. With respect to psychological processes, such as retirement decision making and adjustment and life-span development, various conceptual models have already been introduced by previous literature (e.g. Wang and Shultz 2010; Feldman and Beehr 2011; Wang et al. 2011; Löckenhoff 2012). Regarding the labour market aspect of retirement, i.e. individuals being in and moving between different labour market statuses, previous theoretical models have placed much more focus on the determinants and/or consequences of retirement (Beehr 1986; Wang and Shultz 2010; Hasselhorn and Apt 2015; Fisher et al. 2016) than conceptualization of retirement itself. The conceptual framework introduced in this article focuses specifically on the processes of labour market participation relating to retirement. In the remaining article we always refer to this approach when addressing retirement. We use the term “work-to-retirement process” to cover the wide range of elements that can be perceived as relating to individuals being in the process of moving from work to retirement. The elements may include e.g. early, late and partial retirement, statutory retirement, work disability and unemployment paths to retirement, or different types of bridge employment. We use the term process because the addressed phenomenon may well consist of multiple successive labour market events instead of a single transition from work to retirement.

The previous literature has widely addressed various elements related to the labour market dynamics of the work-to-retirement process (see the following section). However, a conceptual framework that comprehensively addresses the connections between the different elements is currently lacking. We believe that a novel conceptual framework accounting for the complex labour market dynamics of the work-to-retirement process would help researchers to determine what elements of the process are of interest to their research question and which of these elements are captured with the measures that they are using and developing. The conceptual framework would thus enhance well-defined, precise and comparable measurement of the work-to-retirement process.

The intention of our conceptual framework is to help the research community that is interested in the scientific investigation of the work-to-retirement process, to better address the complexity in later-life labour market dynamics. The work was done as a part of the EU-funded project OMEGA-NET (Network on the Coordination and Harmonisation of European Occupational Cohorts; omeganetcohorts.eu). One of the aims of this network is to harmonize concepts and measures relevant to occupational health, including those related to the work-to-retirement process.

### Previous conceptualization of retirement

Diversity of the retirement concept has been well acknowledged in the literature. Beehr (1986) distinguished between different forms of the act of retirement such as early vs. on-time retirement and part-time vs. complete retirement. Hasselhorn and Apt (2015, p. 21) emphasized the fragmented nature of retirement, which may include different elements such as “bridge employment, second career, (part time-) disability retirement, unemployment, unsalaried periods, part time leave, part time pension, early retirement, work while drawing pension, salaried while not working, “regular” retirement, work past retirement age, un-retirement, etc.”.

Cahill et al. (2015) took a dynamic approach when modelling the various labour market pathways to retirement. They considered retirement as a continuum in the time between full-time career employment and complete labour force withdrawal. Between the two ends of the continuum there may be (1) reductions in the work hours, which may occur through phased retirement (a reduction in work hours without changing the employer), (2) bridge employment (taking a new job after career employment) or (3) re-entry into the labour market after an initial departure.

Beehr and Bennett (2015) defined bridge employment more broadly to include “any paid work after retiring, by any definition of retirement”. They also presented a typology of bridge employment with four dichotomies—career jobs versus noncareer jobs, immediate vs. delayed, steady versus intermittent, and self-employed vs. other employed—being crossed with each other in a matrix resulting in 16 different categories. All in all, a commonly addressed feature of bridge employment is that it is linked to a reduction in work duties, not only in terms of reduced work hours, but also in terms of reduced physical and mental work demands and more temporal and spatial flexibility in performing the work tasks (Beehr and Bennett 2015; Zhan 2016).

Empirical studies have also defined retirement in very different ways. Hakola (2003) highlighted this diversity by reviewing how retirement has been addressed based on different elements or their combinations. The elements included subjective assessment of retirement status, hours worked, wage earnings and different definitions based on pension receipt. Further distinctions by full or partial retirement and different retirement types based on e.g. old-age, disability or unemployment, add to the diversity in how retirement is understood.

Denton and Spencer (2009) conducted a literature review on how retirement has been addressed in previous studies. They categorized the findings into eight categories: (1) non-participation in the labour force, (2) reduction in work hours and/or earnings, (3) work hours or earnings below a specified threshold, (4) receipt of retirement income, (5) leaving the main career employer, (6) change of career or employment later in life, (7) self-assessed retirement and (8) combinations of the above. Although the findings indicated large variation in the ways of addressing retirement, all categories were dichotomous by nature, i.e. indicating that a person was either “retired” or “not retired”.

In various recent studies, the work-to-retirement process has been addressed in more dynamic ways than by using a single dichotomous change in status. Changes in labour market status over repeated measurements, covering elements inherent to the work-to-retirement process, have been examined through pre-defined (Boissonneault and de Beer 2018) or empirically driven (Fasang 2010; Tang and Burr 2015; Calvo et al. 2018; Riekhoff 2018) pathways. In other words, labour market status, such as full- or part-time employment, unemployment, disability or other benefit recipiency, partial or full retirement, or being otherwise outside the labour force, have been followed up over an age span of older individuals approaching typical retirement ages in order to demonstrate typical pathways of the work-to-retirement process. These pathways have included e.g. early, normal or late retirement, abrupt or gradual retirement with part-time employment often playing a role, labour market patterns predominantly including unemployment, disability or inactivity, or ones showing diverse patterns. Moreover, changes in labour market status have been examined in terms of observed (Leinonen et al. 2020) or expected (Shiri et al. 2021) time spent in different statuses when approaching retirement age, with working life expectancy as the most commonly used estimate. However, calculations have also been made for the time spent in e.g. unemployment, work disability, pre- and post-retirement employment, as well as different types of retirement.

Irrespective of whether retirement is addressed in a dichotomous or a more complex way, conceptual clarity is called for in future studies. The existing literature has already introduced a broad range of elements related to the labour market dynamics of the work-to-retirement process. However, a conceptual framework providing a comprehensive overview of the potential elements of the process and their connections with each other is lacking so far. Therefore, we want to introduce a conceptual framework of the work-to-retirement process.

### Conceptual framework of the work-to-retirement process

Our conceptual framework aims to incorporate important elements that have been addressed in previous conceptualizations of the work-to-retirement process, from the point of view of labour market dynamics. Moreover, the framework aims to elaborate how these elements are connected to each other. Our purpose is not to provide recommendations for how to define “work”, “retirement” or particular elements of the work-to-retirement process, but rather to introduce a framework addressing the complexity of the process.

**Fig. 1:**
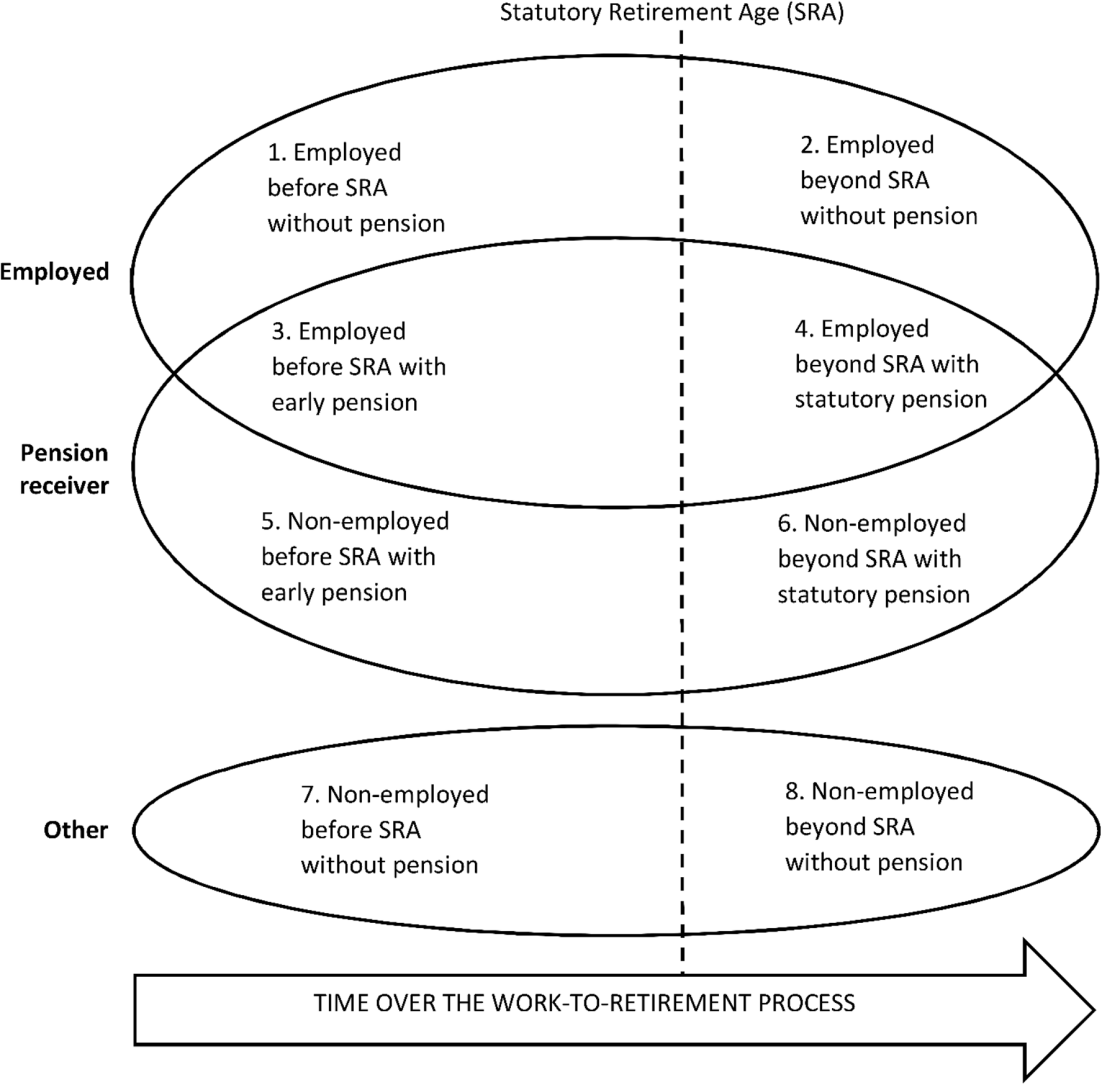
Conceptual framework of the work-to-retirement process *Notes*: The ovals represent main elements of labour market participation (employed, pension receiver and other) and the vertical dashed line represents the statutory retirement age (SRA). The numbered areas indicated by the partly overlapping main elements and the SRA represent further elements of the work-to-retirement process

The conceptual framework consists of two levels; (1) a basic framework, and (2) its specifications. The basic framework is visualized in Fig. 1. The ovals represent the main elements of labour market participation, including employment, pension receipt, and other, i.e. neither being employed nor receiving a pension. The ovals representing employment and pension receipt are overlapping, reflecting the possibility of their co-occurrence. The vertical line crossing the ovals represents the statutory retirement age. The definition of the statutory retirement age may depend considerably on the context and may vary both between and within countries. In some countries it may e.g. be flexible instead of fixed and there may be variations between different groups of employees depending on their type of work or occupation. Also, the statutory retirement age has been subject to change in many countries over the past years and will be so in coming years. The areas indicated by the main elements of labour market participation and the statutory retirement age are numbered to represent further elements of the work-to-retirement process. The horizontal arrow at the bottom of the figure represents time over the life course, during which an individual can move vertically between the different categories of labour market participation. We do not specify a particular age span for the work-to-retirement process, but this can be expected to mostly pertain to ages relatively close to the statutory retirement age. Note that the sizes of the areas do not reflect how common these elements are in the work-to-retirement process.

Area 1 represents what is typically considered as “normal” employment, i.e. being employed before reaching the statutory retirement age and not receiving any pension benefits. Employment may be continued beyond the statutory retirement age without starting to draw the pension (area 2). Areas 3 and 4 represent employment while receiving early and statutory pensions, respectively. Areas 5 and 6 represent early and statutory pension receipt respectively, without being employed. Finally, a person approaching statutory retirement age can be neither employed nor receive a pension (areas 7 and 8).

**Fig. 2:**
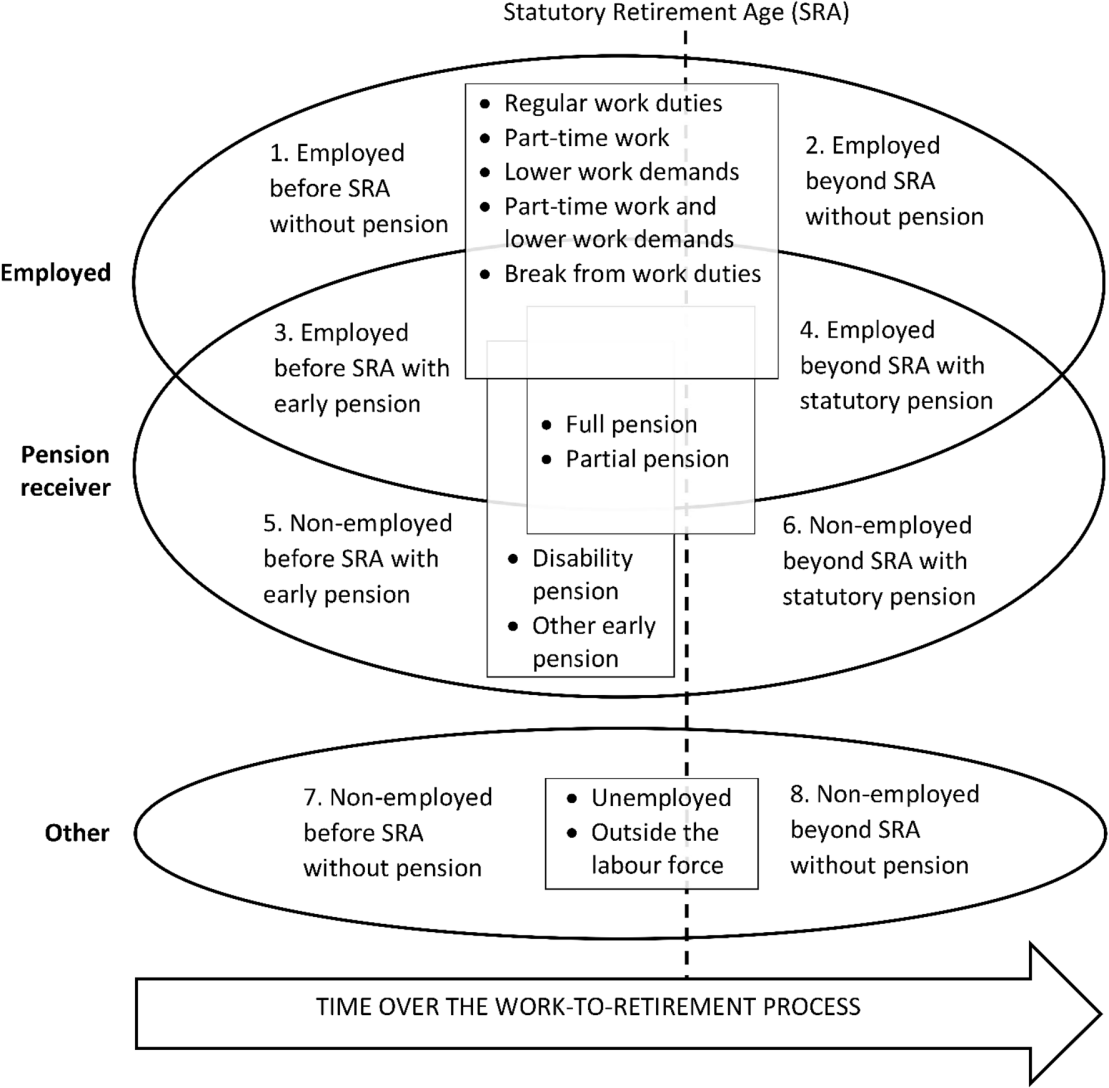
Conceptual framework of the work-to-retirement process with a model example of specifications *Notes*: The ovals represent main elements of labour market participation (employed, pension receiver and other) and the vertical dashed line represents the statutory retirement age (SRA). The numbered areas indicated by the partly overlapping main elements and the SRA represent further elements of the work-to-retirement process. A model example of specifications of these elements is presented within the boxes traversing these areas.

Another level can be added to the basic framework by including specifications of the various elements of the work-to-retirement process. We provide a model example of such specifications, presented in the boxes that traverse the areas representing the elements in Fig. 2. Employment, with or without receiving pensions, may be with regular or reduced work duties. Regular work duties may mean e.g. duties that have been typical during one’s working career. In some contexts, it may be possible to receive pensions even while being employed in regular work duties, if e.g. a person has had multiple jobs with different pension arrangements or if there is no automatic linkage between work cessation and pension receipt in the retirement system. A person can also be employed with reduced work duties in terms of working part time, having lower demands of the work tasks, or both. There may also be breaks from performing work duties due to sickness or other reasons, even if the employment relationship is continued.

Both early and statutory pension may be either full or partial. Early retirement may be further categorized into disability and other early retirement. A person who neither is employed nor receives a pension may be unemployed or outside the labour force for other reasons than pension receipt.

With the eight elements of the basic framework and different combinations of the model specifications, the framework produces a large number of categories representing different but overlapping elements of the work-to-retirement process. To avoid overcomplicating the framework, the number of model specifications was not extended further. However, the model specifications can be further elaborated to incorporate additional specifications to the framework. Employment participation and benefit receipt are partly driven by individual choices, but they further depend on the prevailing social security system and labour market context, which should be taken into account when including additional specifications to the framework.

For example, countries may have specific features regarding pensions and other benefits that need to be considered in the additional specifications. Some countries have specific types of early retirement pensions that may need to be distinguished from the more universal ones. For instance, eligibility to early retirement depends on the duration of pension-insured employment in countries such as Luxemburg, Belgium and Germany, and on the amount of pension savings in Norway. Furthermore, in the Scandinavian countries with relatively generous social security systems, disability retirement has been a particular type of early retirement pension, options for receiving partial pensions have been provided, and drawing a pension has typically been closely tied to the process of retirement. In some other countries, instead, people with disabilities may receive benefits that are not classified as pensions, there may be lack of options to draw partial pensions, and eligibility to public pensions may play a smaller role in the retirement process with e.g. private savings being relied upon. Moreover, for countries with a flexible pension age such as Finland and Norway, setting the statutory retirement age in the framework is less clear cut. This could be solved by e.g. applying the lower age limit, the average or most typical age for starting to draw the flexible pension or by adding a vertical specification to the model representing the range within the statutory pension age.

Further additional specifications may be relevant for examining different types of work. Employment with reduced work duties may occur in the current job, or with a new employer, occupation, and/or industry. They may also occur with changes between wage-employment and self-employment. Overall changes in employment, without simultaneous reductions in work duties, could in some cases also be perceived as being a part of the work-to-retirement process. However, long-term career jobs can no longer be expected to be the norm and career changes may also occur at later stages of working life (Riekhoff 2018; Garthe and Hasselhorn 2021). Later-life career changes may therefore not necessarily reflect the process of withdrawing from the labour market. They may in some cases reflect renewed career aspirations or upward career mobility.

There may also be relevant additional specifications for reasons not to work. Breaks in performing work duties may occur for various reasons, e.g. due to sickness absence, other leaves from work, or zero-hour contracts with no work. A person may also be outside the labour force for various reasons, e.g. due to care-giving responsibilities, other unpaid work, or leisure activities.

All in all, the conceptual framework highlights the complexity of the work-to-retirement process, bringing forth its multifaceted, multiphased and multidirectional features. The process may be multifaceted, with e.g. possibilities of combining employment and pension receipt (areas 3 and 4), being non-employed due to other reasons than being a pensioner (areas 7 and 8), and continuing employment beyond the statutory retirement age (areas 2 and 4). The process may also be multiphased, with e.g. an initially employed individual (area 1) first starting to receive an early pension while still continuing in employment (area 3) and later withdrawing completely from employment while drawing a statutory pension (area 6). Finally, the work-to-retirement process may be multidirectional if e.g. an initially employed individual (area 1) exited employment while starting to receive an early pension (area 5) and later stopped receiving this pension while returning to employment (area 1).

### Applying the conceptual framework to guide measurement of the work-to-retirement process

The presented conceptual framework of the work-to-retirement process, highlighting the complexity of later-life labour market dynamics, implies that the phenomenon cannot be captured by a single question in surveys or a single source of information in the registers. Instead, combinations of information are preferred. How the complexity of the process can be measured, depends on the study context, research questions, available data, and used methods. It is therefore not feasible to recommend a harmonized measure of the work-to-retirement process that would fit all study circumstances.

Our conceptual framework of the work-to-retirement process can nevertheless help researchers in several ways. Firstly, it can help in the *development of measures* by demonstrating the broad range of elements of the process that may be important to account for. As such it may help plan new data collection by providing a check list for capturing all relevant elements. Secondly, it can help *report and interpret findings of studies* by increasing awareness on what elements of the complex process are captured by the used measures. Thirdly, it can help *compare studies* by elaborating the extent to which the used measures overlap. Fourthly, it can help *harmonize future measures* by providing an outline that can be utilized in the assessment of how the different elements of the process are comparable in different contexts.

Together with information on the statutory retirement age, the main elements of the conceptual framework are based on very basic information of labour market participation, including employment, pension receipt and their counterpart. The use of the framework in its simplest form therefore requires very little data. At the same time, the framework is flexible to the user, providing the possibility to add various specifications depending on the study context and aims. Flexibility of the framework also ensures that it can be adapted according to future changes in pension policies and labour market conditions.

It should be noted that it is not always possible or even relevant to capture all elements of the conceptual framework of the work-to-retirement process. We nevertheless believe that the framework—applied with different levels of specificity—can help guide measurement of the complex work-to-retirement process.

There is, however, a certain limitation to using the framework. Although it can be applied both when using self-reported or objective measures of labour market participation of the study participants, it is less applicable to measures that are based on single self-assessed notions of “being retired”. All categories of the framework include labour market statuses based on which some individuals could consider themselves as retired. Accordingly, individuals in one category of the framework can have different notions of whether or not they are retired. One way of including subjective notions of retirement to the framework could be including them as a further specification, but even then, information on the main elements of labour market participation would still be required.

## Conclusions

The large diversity in how the work-to-retirement process has been conceptualized and measured has likely influenced the findings of studies on causes, consequences and patterns of retirement. Accounting for the complex labour market dynamics of the work-to-retirement process in a comprehensive way helps to elaborate what is actually addressed when investigating “retirement”. Our conceptual framework can be utilized to enhance well-defined, precise and comparable measurement of the work-to-retirement process in studies.
